# Supplementary material for: Farmers’ Risk Perception on Climate Change: Transhumance vs. Semi-Intensive Sheep Production Systems in Türkiye
Source: Animals (Basel). 2022 Aug 6;12(15):1992. doi: 10.3390/ani12151992 (PMC9367526; doi:10.3390/ani12151992)
Supplement: Supplementary file 1 [file animals-12-01992-s001.zip › animals-1787616-supplementary.pdf]

Supplementary Table S1: Survey questions

Age of framer	
household	
Education	1:Primary<br>2: Secondary<br>3: Lyceum<br>4:College
How many years of farmer	1:<10<br>2: >10
Agricultural income	>20000TL<br><20000TL
Income other than Agriculture	0: N<br>1: Y
Whether children work	0:N<br>1:Y
Flock size	1:≤100<br>2:100-200<br>3:>200
Grazing land	1:<20 da<br>2: 20-50 da<br>3:>50 da
Decision taking method	1:Personal<br>2:together with the family<br>3: together with the environmental impact
Knowledge about climate change	0:N<br>1:Y
Climate knowledge recourse	1: N<br>2:TV<br>3:Internet<br>4: TV and internet

	Mean	1	2	3	4	5
Decrease in productivity						
Change in temperature						
change in precipitation						
Incidence of natural disasters						
Increase in workload						
Increase in vaccine and costs						
Increase in water usage and cost						
Difficulty in paying the loan						
Decrease in meat and milk quality						
Decrease in flock reproduction parameters						
Change in migration route						
Change of migration time						
Decrease in natural grassland						
1= strongly disagree, 2= disagree, 3= no idea, 4= agree, 5= strongly agree						

	Yes	No
Seasonal feed ratio change		
Use of feed additives		
Increase in water consumption		
Using of cooling system		
Better preservation of feeds		
Diversity of forage crops		
Providing shade in the extreme heat		
Selling animals to buy feed		
Insuring the animals		
Considering crop production		
Rainwater harvesting		
